# Association between hemoglobin glycation index and poor outcome after endovascular thrombectomy in acute ischemic stroke

**DOI:** 10.3389/fnagi.2025.1533584

**Published:** 2025-02-04

**Authors:** Yan Yang, Mei Liu, Shungui Huang, Chen Zhu, Guangzong Li, Bin Wang, Xiaojing Luo, Lingwen Zhang, Weizheng Song

**Affiliations:** ^1^Department of Neurology, The Sixth People’s Hospital of Chengdu, Chengdu, China; ^2^Department of Neurology, Affiliated Hospital of Panzhihua University, Panzhihua, China

**Keywords:** hemoglobin, stroke, thrombectomy, HbA1c, outcome

## Abstract

**Background:**

The prognostic significance of hemoglobin glycation index (HGI) on acute ischemic stroke (AIS) patients treated with endovascular thrombectomy (EVT) remained unclear. This study aimed to investigate the association between HGI and the risk of poor outcome after EVT.

**Methods:**

We retrospectively enrolled AIS patients with large vessel occlusion in the anterior circulation treated with EVT from a multicenter study. Poor outcome was defined as a modified Rankin scale score > 2 points at 90 days after EVT. We used multivariable logistic regression models to investigate the association between HGI and poor outcome. We employed the restricted cubic spline curve to visualize the association between HGI and the risk of poor outcome after EVT.

**Results:**

Among the 403 enrolled patients (median age, 72 years; 63.8% male), a total of 198 (49.1%) patients had poor outcome at 90 days. The restricted cubic spline curve showed that there was a U-shape relationship between HGI and the risk of poor outcome (*P* for non-linearity < 0.001). After divided patients into three groups based on HGI tertiles, HGI (tertile 1 vs. 2) was significantly associated with poor outcome [odds ratio (OR), 3.84; 95% confidence interval (CI), 2.08–7.22; *P* < 0.001] and early neurological deterioration (OR, 3.11; 95% CI, 1.55–6.44; *P* = 0.002) in multivariable analyses. Adding HGI into models improved the discriminative ability for poor outcome (*P* < 0.001).

**Conclusion:**

In conclusion, our study identified a U-shaped relationship between HGI and poor outcome, with low HGI levels significantly associated with poor outcome after EVT.

## Introduction

Endovascular thrombectomy (EVT) has become the gold standard for managing acute ischemic stroke (AIS) caused by large vessel occlusion (LVO), demonstrating significant efficacy in reducing disability and mortality (Goyal et al., 2016). Both clinical trials and real-world evidence have confirmed its effectiveness, even in patients with extended treatment windows or large infarcts (Jovin et al., 2015; Albers et al., 2018; Nogueira et al., 2018; Huo et al., 2023; Sarraj et al., 2023). However, despite successful recanalization, more than half of these patients fail to achieve functional independence, which is influenced by factors such as the stroke severity and stress hyperglycemia (Linfante et al., 2016; van de Graaf et al., 2022; Wang et al., 2023). This underscores the urgent need to investigate potential predictors of poor functional recovery and to facilitate the early identification of AIS patients at high risk of poor outcome after EVT.

Glycosylated hemoglobin (HbA1c) is a widely utilized biomarker that reflects the average blood glucose levels over 3 months. It plays an important role in diagnosing diabetes mellitus and monitoring glucose-lowering therapies (Genuth et al., 2003). HbA1c levels majorly depend on mean plasma glucose levels, accounting for 60–80% of its variability (Herman et al., 2007; Nayak et al., 2019). However, the remaining variation is attributed to factors independent of glucose levels, such as physiological differences and genetic factors. To address this residual variation, Hempe et al. (2002) introduced the hemoglobin glycation index (HGI). HGI provides a quantitative measure of the difference between observed and predicted HbA1c levels (Delpierre et al., 2006). The predicted HbA1c is derived using linear regression models based on the fasting blood glucose (FBG) and observed HbA1c levels. Previous studies had reported the prognostic significance of HGI in various diseases. A U-shaped relationship between HGI and all-cause mortality was observed in patients with critical coronary heart disease (Wei et al., 2024). The *post-hoc* analyses of a randomized clinical trial revealed that low HGI levels were associated with lower risk for cardiovascular mortality in diabetes patients with acute coronary syndromes (van Steen et al., 2017). For acute ischemic stroke patients with diabetes, intermediate tertiles of HGI were linked to a lower probability of poor outcome (Pan et al., 2018). Additionally, in the general population, elevated HGI levels were associated with an increased risk of stroke onset (Wang et al., 2022). However, the relationship between HGI and clinical outcomes in AIS patients treated with EVT remained unexplored.

Hence, we performed this multicenter study to investigate the potential association between HGI and poor outcome in AIS patients treated with EVT.

## Materials and methods

Data that support the findings of this study were available from the corresponding authors upon reasonable request.

### Study population

**FIGURE 1:**
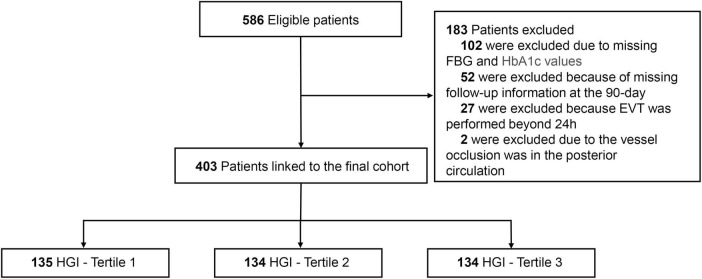
The flow chart of the study. EVT, endovascular thrombectomy; FBG, fasting blood glucose; HGI, hemoglobin glycation index.

This multicenter retrospective study was conducted at the Sixth People’s Hospital of Chengdu and Affiliated Hospital of Panzhihua University from June 2019 to June 2024. The flow chart of this study was shown in Figure 1. Eligible patients met the following criteria: (1) age ≥ 18 years; (2) diagnosis of AIS with LVO in the anterior circulation (internal carotid artery, M1 or M2 segment of the middle cerebral artery); (3) underwent EVT within 24 h of symptom onset; (4) Alberta Stroke Program Early CT Score (ASPECTS) score ≥ 3; (5) National Institute of Health Stroke Scale (NIHSS) score ≥ 6; and (6) pre-stroke modified Rankin Scale (mRS) score < 2. Patients with incomplete baseline FBG or HbA1c data and follow-up information were excluded. The study was approved by the ethics committee of each center and adhered to the ethical standards of the 1964 Helsinki Declaration and subsequent amendments. Written informed consent was waived due to the retrospective nature of this study.

### Data collection and measures

The following variables were collected in this study: demographic data, vital signs, medical history, laboratory test results, procedural parameters, stroke subtypes as defined by the Trial of ORG 10172 in Acute Stroke Treatment (TOAST) classification (Adams et al., 1993), stroke severity measured by the NIHSS score (Brott et al., 1989), and the extent of ischemia assessed using the ASPECTS score (Barber et al., 2000). Drinkers were defined as those who have consumed any type of alcohol within the past 12 months. Brain computed tomography (CT) or magnetic resonance imaging (MRI) were routinely performed at admission and repeated within 24 h post-procedure or in cases of neurological deterioration. Radiological images were independently reviewed by two experienced neurologists blinded to the study details. EVT procedures were performed by experienced neuro-interventionists utilizing stent retrievers, aspiration thrombectomy, or a combination of these techniques according to the Chinese Stroke Association guidelines on reperfusion therapy for acute ischemic stroke 2024. Recanalization was defined as achieving a modified Thrombolysis in Cerebral Ischemia (mTICI) score of 2b/3 on the final angiographic images. Early neurological deterioration (END) was defined as an increase of ≥ 4 points in the NIHSS score between baseline and the evaluation conducted at 48 h after EVT (Kim et al., 2019). Symptomatic intracranial hemorrhage (SICH) was defined as the occurrence of intracranial hemorrhage (ICH) according to the Heidelberg Bleeding Classification (von Kummer et al., 2015).

### HGI calculation

**FIGURE 2:**
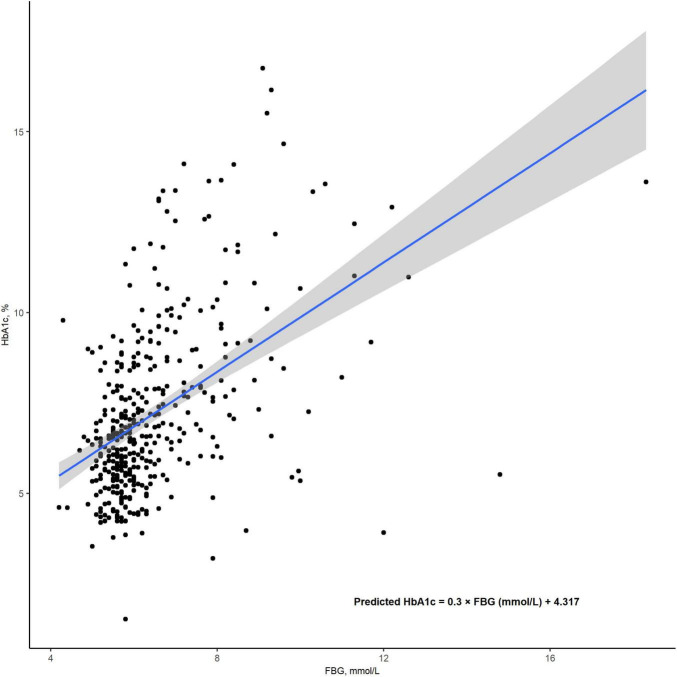
The correlation between FBG and HbA1c levels. HGI was calculated as the difference between the measured HbA1clevels and the predicted HbA1c levels, using the formula: (HGI = measured HbA1c - predicted HbA1c). The predicted HbA1c was derived through a linear regression model, calculated as: predicted HbA1c = 0.3 × FBG (mmol/L) + 4.317 (*r*^2^ = 0.226; *P* < 0.001). FBG, fasting blood glucose; HbA1c, Hemoglobin A1c; HGI, hemoglobin glycation index.

All blood samples were collected immediately upon admission. HGI was calculated as HbA1c - 0.3 × FBG (mmol/L) + 4.317 (*r*^2^ = 0.227; *P* < 0.001; Figure 2). HGI values were then categorized into tertiles as follows: tertile 1 (< -0.524), tertile 2 (-0.524 to 0.055), and tertile 3 (> 0.055).

### Functional outcome

Functional outcomes were assessed at 90 days after EVT using the mRS score. Evaluations were conducted by two trained neurologists blinded to the study design through standardized interviews. Poor outcome was defined as a score > 2 on the mRS score at 90 days.

### Statistical analysis

Continuous variables with normal distributions were expressed as mean ± standard deviation (SD) and compared using the *t*-test. Non-normally distributed continuous variables were presented as medians with interquartile ranges (IQR) and analyzed using the Mann-Whitney U test. Categorical variables were expressed as counts and percentages [*n* (%)] and compared using Chi-square tests or Fisher’s exact tests, depending on the data distribution. For comparisons across multiple groups, trend tests were applied. Missing values were handled with multiple imputations via chained equations.

We employed multivariable logistic regression models to evaluate the association between HGI tertiles and clinical outcomes after EVT, including poor outcome, END, ICH and SICH. Model 1 was adjusted for age and sex. Model 2 was additionally adjusted for smoke, drink, hypertension, diabetes mellitus, hyperlipidemia, coronary heart disease, and atrial fibrillation. Model 3 was covariates with *P*-value < 0.1 in the univariable analyses, inflammatory markers and oxidative stress indicators after back-ward section. Variables in the final step were age, diabetes mellitus, puncture to reperfusion (PTR), baseline NIHSS score, baseline ASPECTS score, number of attempts, HbA1c, white blood cell counts, neutrophil count and lymphocyte count. The results of logistic regression models were presented as odds ratio (OR) and 95% confidence interval (CI).

To visualize the relationship between HGI and poor outcome, we applied a restricted cubic spline analysis with three knots placed at the 10th, 50th, and 90th percentiles, adjusting for variables included in Model 3. We utilized the receiver operating characteristic (ROC) curve to evaluate the discriminative performance of the non-linear term of HGI in predicting poor outcome after EVT. Additionally, we assessed the improvement in discriminative performance by incorporating HGI tertiles into the three predictive models, using the net reclassification improvement (NRI), integrated discrimination improvement (IDI) metrics, Brier score, F1 score, and accuracy. In sensitivity analyses, we performed multivariable logistic regression models after excluding patients with failed recanalization. We also recalculated the HGI value by the formula developed by Li et al. (2022). and reperformed the analyses. Furthermore, we conducted subgroup analyses stratified by age, sex, hypertension, diabetes mellitus, atrial fibrillation, IVT, and NIHSS score, which explored potential interactions between HGI tertiles and these variables, ensuring the robustness of the findings.

All statistical analyses were conducted with R statistical software version 4.2.1. (R Foundation, Vienna, Austria), and a two-sided *P*-value < 0.05 was considered to be statistically significant.

## Results

### Baseline characteristics

**FIGURE 3:**
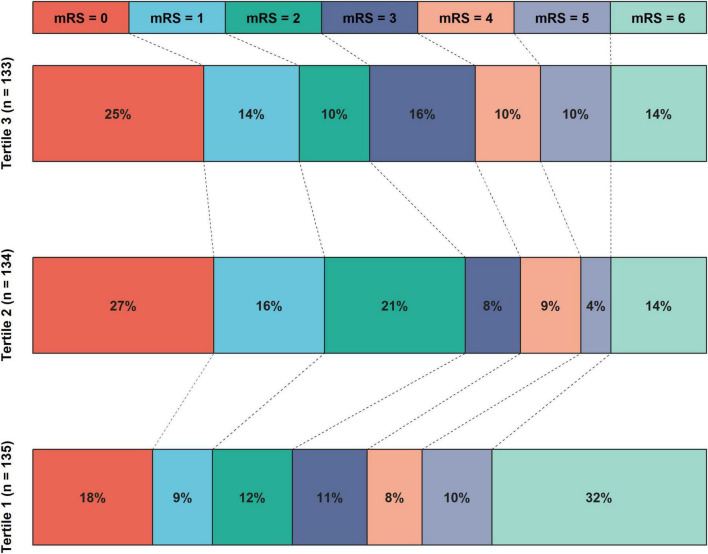
The distribution of proportions of 90 days mRS scores after EVT according to HGI tertiles. The mRS score distribution was depicted using various color blocks, with mRS = 0 signifying the best prognosis and mRS = 6 indicating the worst. The histogram revealed that patients in tertile 1 of the HGI exhibited the highest risks of poor outcomes, while those in the tertile 2 had the lowest risks of poor outcomes. EVT, endovascular thrombectomy; HGI, hemoglobin glycation index; mRS, modified Rankin Scale Score.

A total of 403 patients were included in this study after the exclusion of 183 patients due to missing information or not meeting the inclusion criteria. The study population had a median age of 72 years [IQR (64, 79)] and 257 (63.8%) male patients. Among the enrolled patients, 160 (39.7%) were treated with IVT, 195 (48.4%) had M1 segment occlusion, and 381 (94.5%) achieved successful recanalization. Regarding clinical outcomes, 198 (49.1%) had poor outcome, 79 (19.6%) experienced END, 99 (24.6%) developed ICH, and 52 (12.9%) had SICH after EVT. After divided patients into three groups based on HGI tertiles, patients with tertiles 1 had lower HbA1c, international normalized ratio, and triglyceride, lower proportions of hypertension, diabetes mellitus, coronary heart disease, and stent implement, higher FBG and NIHSS score, higher proportions of cardioembolic stroke and M1 segment occlusion, higher risks of ICH, END and poor outcome (all *P* < 0.05; Figure 3 and Table 1).

**TABLE 1:** Baseline characteristics of the study population according to hemoglobin glycation index (HGI) tertiles.

Characteristics	Total (*n* = 403) (-2.940, 9.927)	Tertile 1 (*n* = 135) (< -0.524)	Tertile 2 (*n* = 134) (-0.524, 0.055)	Tertile 3 (*n* = 134) (> 0.055)	*P*-value
Age, years [median (IQR)]	72.0 (64.0, 79.0)	74.0 (64.5, 83.0)	70.5 (63.0, 78.0)	71.0 (64.2, 77.0)	0.101
Male, *n* (%)	257 (63.8)	83 (61.5)	84 (62.7)	90 (67.2)	0.594
Height, cm [median (IQR)]	165.0 (160.0, 170.0)	165.0 (160.0, 170.0)	166.5 (160.0, 170.0)	165.0 (160.0, 170.0)	0.742
Weight, kg [median (IQR)]	67.0 (60.0, 75.0)	65.0 (55.0, 75.0)	65.0 (60.0, 75.0)	70.0 (60.0, 75.0)	0.077
SBP, mmHg [mean (SD)]	155 (38.5)	46 (34.1)	50 (37.3)	59 (44.0)	0.231
DBP, mmHg [mean (SD)]	90 (22.3)	25 (18.5)	32 (23.9)	33 (24.6)	0.422
Smoking	153 (38.4)	45 (33.8)	49 (37.1)	59 (44.4)	0.196
Drinking	89 (22.4)	24 (18.0)	32 (24.2)	33 (24.8)	0.340
**Vascular risk factors, n (%)**					
Hypertension	296 (73.4)	86 (63.7)	98 (73.1)	112 (83.6)	0.001
Diabetes mellitus	115 (28.5)	17 (12.6)	22 (16.4)	76 (56.7)	< 0.001
Hyperlipidemia	43 (10.7)	10 (7.4)	16 (11.9)	17 (12.7)	0.316
Coronary heart disease	67 (16.6)	17 (12.6)	19 (14.2)	31 (23.1)	0.044
Atrial fibrillation	119 (29.5)	44 (32.6)	34 (25.4)	41 (30.6)	0.408
**Laboratory data**					
FBG, mmol/L [median (IQR)]	6.6 (5.5, 8.4)	7.2 (6.4, 8.9)	5.9 (5.2, 7.0)	6.7 (5.4, 8.4)	< 0.001
HbA1c, % [median (IQR)]	6.0 (5.6, 6.8)	5.6 (5.3, 6.0)	5.8 (5.6, 6.2)	7.2 (6.4, 8.4)	< 0.001
TC, mmol/L [median (IQR)]	4.0 (3.4, 4.8)	4.0 (3.4, 4.5)	4.1 (3.4, 4.8)	4.0 (3.4, 4.9)	0.497
TG, mmol/L [median (IQR)]	1.0 (0.7, 1.4)	0.9 (0.7, 1.2)	1.1 (0.8, 1.5)	1.1 (0.8, 1.5)	0.001
INR, [median (IQR)]	1.0 (1.0, 1.1)	1.0 (1.0, 1.1)	1.0 (0.9, 1.1)	1.0 (1.0, 1.1)	0.043
WBC, 10^9^/L, [median (IQR)]	9.2 (7.2, 12.3)	9.3 (7.4, 12.2)	9.2 (7.4, 11.8)	9.1 (7.0, 12.8)	0.881
Neutrophil count, 10^9^/L, [median (IQR)]	7.8 (5.6, 10.4)	8.0 (5.6, 10.7)	8.0 (5.7, 9.9)	7.3 (5.5, 11.2)	0.835
Lymphocyte count, 10^9^/L, [median (IQR)]	1.1 (0.7, 1.5)	1.1 (0.7, 1.5)	1.0 (0.7, 1.4)	1.1 (0.7, 1.6)	0.776
C-reactive protein, [median (IQR)]	7.5 (2.5, 22.5)	7.0 (2.4, 25.9)	7.3 (3.1, 16.7)	8.1 (2.5, 25.3)	0.892
Interleukin-6, pg/mL, [median (IQR)]	18.0 (8.9, 54.8)	13.0 (6.4, 36.6)	20.7 (8.9, 59.0)	19.1 (9.5, 55.7)	0.182
Uric acid, mmol/L, [median (IQR)]	311.0 (242.0, 386.2)	323.0 (257.2, 410.4)	297.9 (229.0, 379.8)	305.6 (244.6, 379.8)	0.165
**TOAST (%)**					0.015
LAA	178 (44.2)	45 (33.3)	67 (50.0)	66 (49.3)	–
CE	189 (46.9)	75 (55.6)	53 (39.6)	61 (45.5)	–
Other	36 (8.9)	15 (11.1)	14 (10.4)	7 (5.2)	–
IVT, *n* (%)	160 (39.7)	55 (40.7)	46 (34.3)	59 (44.0)	0.256
**Recanalization outcomes**					
Number of attempts, *n* [median (IQR)]	1.0 (1.0, 2.0)	1.0 (1.0, 3.0)	1.0 (1.0, 2.0)	1.0 (1.0, 2.0)	0.374
mTICI 2b/3, *n* (%)	381 (94.5)	128 (94.8)	125 (93.3)	128 (95.5)	0.712
OTP, min [median (IQR)]	302.0 (190.0, 530.0)	280.0 (190.0, 459.0)	290.5 (186.2, 537.5)	380.0 (231.0, 539.5)	0.062
PTR, min [median (IQR)]	60.0 (42.0, 90.0)	60.0 (42.0, 90.0)	63.0 (43.0, 94.5)	58.0 (43.0, 88.2)	0.483
Baseline NIHSS, score [median (IQR)]	13.0 (10.0, 17.0)	15.0 (12.0, 18.0)	13.5 (10.0, 16.0)	12.0 (8.0, 15.0)	< 0.001
Baseline mRS, score [median (IQR)]	0.0 (0.0, 0.0)	0.0 (0.0, 0.0)	0.0 (0.0, 0.0)	0.0 (0.0, 0.0)	0.212
Baseline ASPECTS, score [median (IQR)]	9.0 (8.0, 9.0)	9.0 (8.0, 9.0)	9.0 (8.0, 9.0)	9.0 (8.0, 9.0)	0.089
**Procedural parameters, n (%)**					
ASITN/SIR 2–3	46 (11.4)	13 (9.6)	19 (14.2)	14 (10.4)	0.458
Stent implement	15 (3.7)	6 (4.4)	5 (3.7)	4 (3.0)	0.819
Intra-arterial thrombolysis	15 (3.7)	6 (4.4)	5 (3.7)	4 (3.0)	0.819
**Occlusion site, *n* (%)**					0.001
ICA	95 (23.6)	27 (20.0)	23 (17.2)	45 (33.6)	–
MCA-M1	195 (48.4)	74 (54.8)	71 (53.0)	50 (37.3)	–
MCA-M2	34 (8.4)	15 (11.1)	14 (10.4)	5 (3.7)	–
T occlusion	79 (19.6)	19 (14.1)	26 (19.4)	34 (25.4)	–
ICH, *n* (%)	99 (24.6)	39 (28.9)	28 (20.9)	32 (23.9)	0.306
SICH, *n* (%)	52 (12.9)	22 (16.3)	12 (9.0)	18 (13.4)	0.194
END, *n* (%)	79 (19.6)	31 (23.0)	17 (12.7)	31 (23.1)	0.047
Poor outcome, *n* (%)	198 (49.1)	83 (61.5)	48 (35.8)	67 (50.0)	<0.001

ASITN/SIR, the American Society of Interventional and Therapeutic Neuroradiology/Society of Interventional Radiology; ASPECTS, the Alberta Stroke Program Early Computed Tomography Score; CE, cardioembolism; DBP, diastolic blood pressure; END, early neurological deterioration; EVT, endovascular thrombectomy; FBG, fasting blood glucose; HGI, HbA1c glycation index; ICA, internal carotid artery; ICH, intracranial hemorrhage; INR, international normalized ratio; IVT, intravenous thrombolysis; LAA, large artery atherosclerosis; MCA, middle cerebral artery; mRS, modified Rankin Scale Score; mTICI, modified Thrombolysis in Cerebral Infarction Score; NIHSS, National Institute of Health Stroke Scale; OTP, from onset to puncture; PTR, from puncture to recanalization; SBP, systolic blood pressure; SICH, symptomatic intracranial hemorrhage; TC, total cholesterol; TG, triglyceride; TOAST, the trial of ORG 10172 in Acute Stroke Treatment classification; WBC, white blood cell count.

### Poor outcome

**FIGURE 4:**
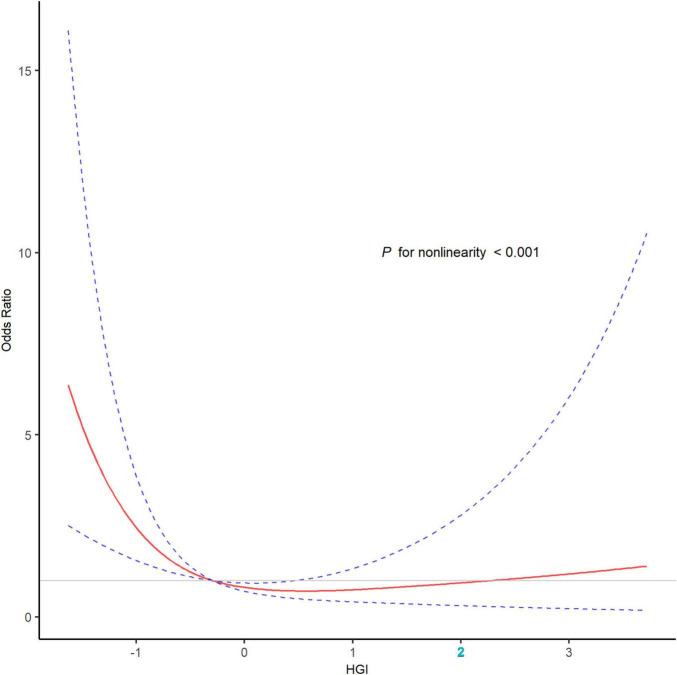
The association of HGI with the risk of poor outcome after EVT in the restricted cubic spline curve. The restricted cubic spline curve was adjusted for variables included in the model 3 with three knots (the 10th, 50th, and 90th percentile), revealed a U-shape relationship between HGI and the risk of poor outcome (*P* for non-linearity < 0.001). The red line represented the odds ratio, and the blue dotted lines represented the 95% confidence intervals. EVT, endovascular thrombectomy; HGI, hemoglobin glycation index.

Patients with poor outcome tended to be older and female, and exhibited more severe stroke symptoms and ischemia. They also have higher rates of adverse clinical conditions such as diabetes mellitus, atrial fibrillation, and cardioembolic stroke. The recanalization of the occluded vessel was more challenging in these patients (*P* < 0.05; Supplementary Table 1). The restricted cubic spline curve showed that there was a U-shape relationship between HGI and the risk of poor outcome (*P* for non-linearity < 0.001, Figure 4). However, the area under the curve revealed that, as a non-linear variable, HGI had a modest predictive ability for poor outcome after EVT (0.628; 95% CI, 0.573–0.683; Supplementary Figure 1).

### Multivariable analyses

In multivariable analyses, HGI (tertile 1 vs. 2) was significantly associated with poor outcome in model 1 (OR, 2.94; 95% CI, 1.74–5.01; *P* < 0.001), model 2 (OR, 3.13; 95% CI, 1.884–5.42; *P* < 0.001), and model 3 (OR, 3.84; 95% CI, 2.08–7.22; *P* < 0.001; Table 2), respectively. Furthermore, adding HGI into three models significantly improved the discriminative ability for poor outcome (model 3: NRI, 0.28; 95% CI, 0.11–0.54; *P* < 0.001; IDI, 0.04; 95% CI, 0.02–0.06; *P* < 0.001; Table 3). HGI also improved the F1 score and accuracy for these models (Supplementary Table 2). Additionally, HGI was associated with END in model 1 (OR, 2.04; 95% CI, 1.08–3.98; *P* = 0.031), model 2 (OR, 2.20; 95% CI, 1.14–4.34; *P* = 0.020), and model 3 (OR, 3.11; 95% CI, 1.55–6.44; *P* = 0.002), respectively. The association between HGI and poor outcomes remained significant in patients with successful recanalization (*P* < 0.05; Supplementary Table 3) and using the new formula (*P* < 0.05; Supplementary Table 4). In sensitivity analyses, no significant interactions were observed between HGI and relevant variables in relation to poor outcome after EVT (all *P* > 0.05; Supplementary Figure 1).

**TABLE 2:** Association between hemoglobin glycation index (HGI) and Clinical Outcomes after endovascular thrombectomy (EVT).

	Model 1		Model 2		Model 3	
**Outcomes**	**OR (95% CI)**	***P*-value**	**OR (95% CI)**	***P*-value**	**OR (95% CI)**	***P*-value**
**Poor outcome**						
HGI tertile 1 vs. 2	2.94 (1.74–5.01)	<0.001	3.13 (1.84–5.42)	<0.001	3.84 (2.08–7.22)	< 0.001
HGI tertile 3 vs. 2	1.97 (1.18–3.32)	0.010	1.34 (0.75–2.37)	0.322	1.37 (0.65–2.90)	0.408
**END**						
HGI tertile 1 vs. 2	2.04 (1.08–3.98)	0.031	2.20 (1.14–4.34)	0.020	3.11 (1.55–6.44)	0.002
HGI tertile 3 vs. 2	2.08 (1.10–4.06)	0.027	2.04 (1.01–4.19)	0.048	1.41 (0.63–3.20)	0.407
**ICH**						
HGI tertile 1 vs. 2	1.49 (0.85–2.63)	0.166	1.46 (0.82–2.60)	0.198	1.60 (0.88–2.91)	0.124
HGI tertile 3 vs. 2	1.20 (0.67–2.14)	0.544	1.09 (0.57–2.06)	0.801	0.94 (0.44–1.96)	0.868
**SICH**						
HGI tertile 1 vs. 2	1.90 (0.91–4.15)	0.095	2.07 (0.97–4.60)	0.065	2.68 (1.22–6.17)	0.017
HGI tertile 3 vs. 2	1.60 (0.74–3.57)	0.233	1.36 (0.59–3.23)	0.476	1.08 (0.41–2.83)	0.873

Model 1: Adjusted for age and sex. Model 2: Additionally adjusted for smoke, drink, hypertension, diabetes mellitus, hyperlipidemia, coronary heart disease, and atrial fibrillation. Model 3: Adjusted for covariates with *P*-value < 0.1 in the univariable analyses, inflammatory markers and oxidative stress indicators after back-ward section. Variables in the final step were age, diabetes mellitus, PTR, baseline NIHSS score, baseline ASPECTS score, number of attempts, HbA1c, WBC, neutrophil count and lymphocyte count. ASPECTS, the Alberta Stroke Program Early Computed Tomography Score; CI, confidence interval; EVT, endovascular thrombectomy; HGI, HbA1c glycation index; ICH, intracranial hemorrhage; NIHSS, National Institute of Health Stroke Scale; OR, odds ratio; PTR, puncture to recanalization; SICH, symptomatic intracranial hemorrhage; WBC, white blood cell count.

**TABLE 3:** Reclassification indexes for hemoglobin glycation index (HGI) and poor outcome after endovascular thrombectomy (EVT).

Models	NRI (95% CI)	*P*-value	IDI (95% CI)	*P*-value
Model 1	0.35 (0.14–0.56)	<0.001	0.04 (0.02–0.06)	<0.001
Model 2	0.37 (0.17–0.55)	<0.001	0.04 (0.03–0.06)	<0.001
Model 3	0.28 (0.11–0.54)	<0.001	0.04 (0.02–0.06)	<0.001

Model 1: Adjusted for age and sex. Model 2: Additionally adjusted for smoke, drink, hypertension, diabetes mellitus, hyperlipidemia, coronary heart disease, and atrial fibrillation. Model 3: Adjusted for age, diabetes mellitus, PTR, baseline NIHSS score, baseline ASPECTS score, number of attempts, HbA1c, WBC, neutrophil count and lymphocyte count. ASPECTS, the Alberta Stroke Program Early Computed Tomography Score; CI, confidence interval; EVT, endovascular thrombectomy; HGI, HbA1c glycation index; IDI, integrated discrimination improvement; NIHSS, National Institute of Health Stroke Scale; NRI, net reclassification index; PTR, puncture to recanalization; WBC, white blood cell count.

## Discussion

In this multicenter study, we identified a significant association between interindividual variation in HbA1c and clinical outcomes in AIS patients treated with EVT. We observed a U-shaped relationship between HGI and the risk of poor outcome after EVT, with low HGI levels significantly linked to poor outcome after EVT. Furthermore, incorporating HGI tertiles into predictive models improved the ability to predict poor outcome in AIS patients treated with EVT. Our findings suggested that HGI may serve as a valuable prognostic biomarker for AIS patients treated with EVT, providing insights for individualized risk stratification and management in clinical practice.

HbA1c has been recognized as an important prognostic marker for AIS patients in previous studies. Elevated HbA1c at admission, indicative of poor long-term glucose control, was associated with adverse outcomes. Diprose et al. (2020) reported that higher admission HbA1c was a significant predictor of poor outcome after EVT for AIS, with each 10 mmol/mol increment in HbA1c reducing the likelihood of achieving functional independence at 90 days. Lattanzi et al. (2016) highlighted that approximately 67% of AIS patients with diabetes had suboptimal glucose control (HbA1c ≥ 7%), and this poor control showed a dose-dependent association with unfavorable outcomes after EVT. HGI was proposed as a measure of the discrepancy between actual and theoretical HbA1c levels, effectively quantifying the degree of glycosylation. HGI could prevent errors associated with relying solely on HbA1c by refining the assessment of glycemic impact (Nayak et al., 2019). Therefore, HGI may serve as a valuable biomarker for predicting prognosis in AIS patients after EVT.

Previous studies had confirmed that HGI was a valuable tool for evaluating the incidence of complications and prognostic outcomes in various diseases. In non-diabetic populations, elevated HGI levels was linked to adverse cardiometabolic profiles, including increased markers of liver damage. Notably, individuals with higher HGI levels were found to have a 1.6-fold increased risk of developing hepatic steatosis compared to those with lower HGI levels (Fiorentino et al., 2017). In the AleCardio trial, low HGI in patients with an acute coronary syndrome and diabetes were at lower risk for cardiovascular mortality (van Steen et al., 2017). In the Diabetes Control and Complications Trial, individuals with higher HGI levels were found to have a significantly increased risk of developing or progressing diabetic complications, such as retinopathy and nephropathy, highlighting the importance of the biological variation in HbA1c in assessing diabetes-related complications (McCarter et al., 2004).

In our study, we identified a U-shaped relationship between HGI and poor outcome following EVT. Similarly, Lin et al. (2024) reported a U-shaped association between HGI levels and cardiovascular outcomes in a cohort of 11,921 patients with diabetes and coronary artery disease. Notably, those in the low HGI group (quantile 1) faced a significantly higher risk of all-cause and cardiovascular-related mortality (Lin et al., 2024). Pan et al. (2018) observed a U-shaped relationship between HGI and the prognosis of AIS patients with diabetes. The results highlighted that patients with moderate HGI levels had the lowest risk of poor outcome, suggesting an optimal range of HGI that minimized adverse events (Pan et al., 2018).

The relationship between HGI and poor outcome after EVT may be attributed to the underlying mechanisms: Glycated hemoglobin is produced through a non-enzymatic intracellular process. Variations in erythrocyte turnover rates and glucose transport mechanisms will destabilize HbA1c levels and increase the level of HGI, which may exacerbate the glycemic environment and increase the risk of poor recovery and complications after EVT (Chalew et al., 2005; Hempe et al., 2013). Previous studies suggested that metabolic instability was independently associated with an increased risk of composite outcome and disability in patients treated with reperfusion therapy (Chen et al., 2024). Our study revealed that patients in the low-tertile HGI group exhibited a higher risk of poor outcome compared to those in the other tertiles. This phenomenon may be linked to stress hyperglycemia, which reduced HbA1c levels below predicted glucose concentrations. Acute glucose fluctuations not only exacerbated neuroinflammation but also promoted the release of neurotoxic and vasoconstrictive factors, which promoted progression after stroke (Monnier et al., 2006; Roberts et al., 2015). Additionally, stress hyperglycemia can accelerate cellular acidosis within the ischemic penumbra, ultimately contributing to larger infarct sizes (Anderson et al., 1999). These mechanisms provide a plausible explanation for the observed U-shaped relationship between HGI and poor outcome in our study.

To the best of our knowledge, this was the first study to investigate the association between HGI and poor outcome after EVT. However, there are several limitations to consider. First, as a retrospective analysis with a limited sample size, this study may be subject to potential confounding factors and limited generalizability. A prospective study was required to verify the accuracy and reliability of the findings. Second, we were only able to collect HbA1c and FBG values at admission. Repeated measurements of HGI over time could provide more comprehensive insights. Third, we calculated HGI using FBG as a proxy for average plasma glucose levels. Although average plasma glucose was a more accurate measure, FBG is more readily obtained in clinical settings and had comparable performance to average plasma glucose levels. Finally, this study included only Chinese patients, which may limit the external validity of our findings.

## Conclusion

In conclusion, our study identified a U-shaped relationship between HGI and poor outcome, with low HGI levels significantly associated with poor prognosis after EVT. These findings highlighted the potential of HGI as a valuable tool for early risk assessment and prognosis in AIS patients treated with EVT. Future research was warranted to further explore and validate the prognostic value of HGI in different populations.

## Data Availability

The raw data supporting the conclusions of this article will be made available by the authors, without undue reservation.
