# Supplementary material for: Association between hemoglobin glycation index and poor outcome after endovascular thrombectomy in acute ischemic stroke
Source: Front Aging Neurosci. 2025 Feb 4;17:1533584. doi: 10.3389/fnagi.2025.1533584 (PMC11832471; doi:10.3389/fnagi.2025.1533584)
Supplement: Supplementary file 1 [file Data_Sheet_1.pdf]

Supplementary materials

Figure S1. ROC Curve for HGI to Predict Poor Outcome after EVT.

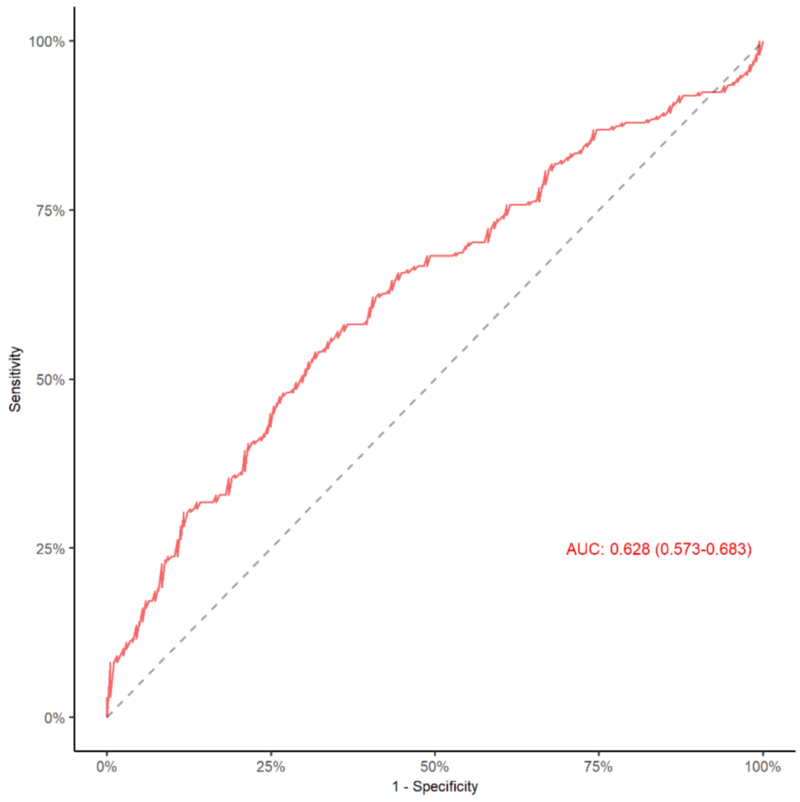

Abbreviations: EVT, endovascular thrombectomy; ROC, receiver operative characteristic; AUC, area under the curve; HGI, hemoglobin glycation index.

Figure S2. Subgroup Analyses for the Association between HGI and Poor Outcome after EVT.

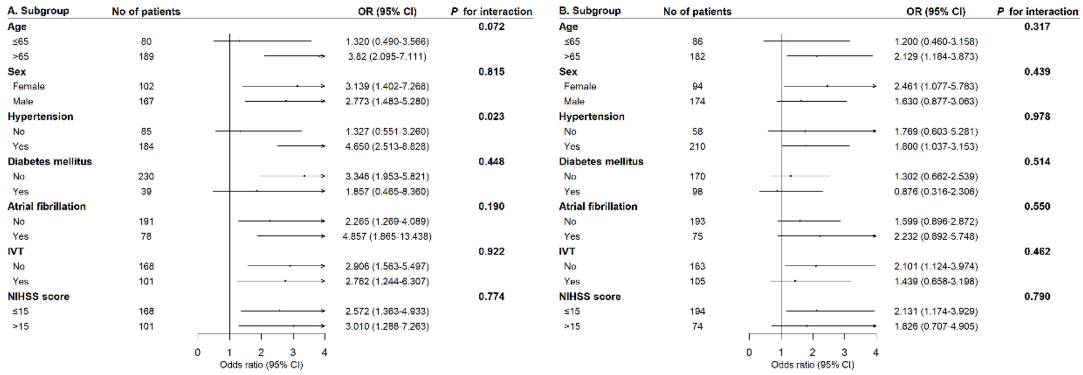

Abbreviations: (A) OR was calculated by comparing participants in tertile 1 with those in tertile 2; (B) OR was calculated by comparing tertile 3 with tertile 2. CI, confidence interval; EVT, endovascular thrombectomy; IVT, intravenous thrombolysis; HGI, hemoglobin glycation index; NIHSS, National Institute of Health Stroke Scale; OR, odds ratio.

Supplementary Table 1. Baseline Characteristics of the Study Population According to Poor outcome after EVT.

Characteristics	With Poor Outcome (n = 198)	Without Poor Outcome (n = 205)	***P* value**
Age, years (median [IQR])	75.0 [69.0, 82.8]	68.0 [59.0, 76.0]	<0.001
Male, n (%)	110 (55.6)	147 (71.7)	0.001
Height, cm (median [IQR])	162.5 [160.0, 170.0]	168.0 [160.0, 170.0]	0.003
Weight, kg (median [IQR])	65.0 [55.0, 75.0]	70.0 [62.0, 75.0]	0.054
SBP, mmHg (mean [SD])	141.5 (23.4)	137.3 (20.7)	0.054
DBP, mmHg (mean [SD])	84.3 (13.8)	84.2 (13.2)	0.942
Smoking	64 (32.3)	91 (44.4)	0.017
Drinking	34 (17.2)	56 (27.3)	0.020
Vascular risk factors, n (%)			
Hypertension	153 (77.3)	143 (69.8)	0.111
Diabetes mellitus	73 (36.9)	42 (20.5)	<0.001
Hyperlipidemia	18 (9.1)	25 (12.2)	0.397
Coronary heart disease	40 (20.2)	27 (13.2)	0.078
Atrial fibrillation	73 (36.9)	46 (22.4)	0.002
Laboratory data			
FBG, mmol/L (median [IQR])	7.3 [6.1, 9.2]	6.1 [5.2, 7.2]	<0.001
HbA1c, % (median [IQR])	6.2 [5.7, 7.1]	5.9 [5.5, 6.6]	0.001
TC, mmol/L (median [IQR])	4.0 [3.4, 4.7]	4.1 [3.4, 4.8]	0.524
TG, mmol/L (median [IQR])	1.0 [0.7, 1.4]	1.0 [0.8, 1.4]	0.618
INR, (median [IQR])	1.0 [1.0, 1.1]	1.0 [1.0, 1.1]	0.086
WBC, 10^9/L, (median [IQR])	9.9 [7.7, 13.4]	9.1 [6.8, 11.4]	0.003
Neutrophil count, 10^9/L, (median [IQR])	8.2 [6.0, 11.7]	7.1 [5.2, 9.7]	0.003
Lymphocyte count, 10^9/L, (median [IQR])	1.1 [0.7, 1.5]	1.1 [0.7, 1.5]	0.817
C-reactive protein, (median [IQR])	7.5 [2.7, 27.9]	7.3 [2.5, 20.2]	0.587
Interleukin-6, pg/mL, (median [IQR])	18.0 [8.1, 55.7]	20.7 [8.9, 54.8]	0.393
Uric acid, mmol/L, (median [IQR])	314.0 [251.6, 386.9]	305.3 [233.0, 385.5]	0.337
HGI tertiles, n (%)			<0.001
Tertile1	83 (41.9)	52 (25.4)	
Tertile2	48 (24.2)	86 (42.0)	
Tertile3	67 (33.8)	67 (32.7)	
TOAST (%)			0.009
LAA	79 (39.9)	99 (48.3)	
CE	107 (54.0)	82 (40.0)	
Other	12 (6.1)	24 (11.7)	
IVT, n (%)	78 (39.4)	82 (40.0)	0.982
Recanalization outcomes			
Number of attempts, n (median [IQR])	2.0 [1.0, 3.0]	1.0 [1.0, 2.0]	<0.001
mTICI 2b/3, n (%)	179 (90.4)	202 (98.5)	0.001
OTP, min (median [IQR])	302.5 [206.2, 491.2]	302.0 [180.0, 540.0]	0.963
PTR, min (median [IQR])	70.0 [50.0, 98.8]	52.0 [40.0, 80.0]	<0.001

Baseline NIHSS, score (median [IQR])	15.0 [12.0, 19.0]	12.0 [8.0, 15.0]	<0.001
Baseline mRS, score (median [IQR])	0.0 [0.0, 1.0]	0.0 [0.0, 0.0]	<0.001
Baseline ASPECTS, score (median [IQR])	8.0 [8.0, 9.0]	9.0 [8.0, 9.0]	<0.001
Procedural parameters, n (%)			
ASITN/SIR 2-3	20 (10.1)	26 (12.7)	0.510
Stent implement	31 (15.7)	51 (24.9)	0.030
Intra-arterial thrombolysis	7 (3.5)	8 (3.9)	1.000
Occlusion site, n (%)			0.069
ICA	58 (29.3)	37 (18.0)	
MCA-M1	89 (44.9)	106 (51.7)	
MCA-M2	15 (7.6)	19 (9.3)	
T occlusion	36 (18.2)	43 (21.0)	
ICH, n (%)	71 (35.9)	28 (13.7)	<0.001
SICH, n (%)	38 (19.2)	14 (6.8)	<0.001
END, n (%)	58 (29.3)	21 (10.2)	<0.001

Abbreviations: ASITN/SIR, the American Society of Interventional and Therapeutic Neuroradiology/Society of Interventional Radiology; ASPECTS, the Alberta Stroke Program Early Computed Tomography Score; CE, cardioembolism; DBP, diastolic blood pressure; END, early neurological deterioration; EVT, endovascular thrombectomy; FBG, fasting blood glucose; HGI, hemoglobin glycation index; ICA, internal carotid artery; ICH, intracranial hemorrhage; INR, international normalized ratio; IVT, intravenous thrombolysis; LAA, large artery atherosclerosis; MCA, middle cerebral artery; mRS, modified Rankin Scale Score; mTICI, modified Thrombolysis in Cerebral Infarction Score; NIHSS, National Institute of Health Stroke Scale; OTP, from onset to puncture; PTR, from puncture to recanalization; SBP, systolic blood pressure; SICH, symptomatic intracranial hemorrhage; TC, total cholesterol; TG, triglyceride; TOAST, the trial of ORG 10172 in Acute Stroke Treatment classification; WBC, white blood cell count.

Table 2. Changes of Brier Score, F1 score and Accuracy after Adding HGI to Models for Poor Outcome after EVT.

Models	Brier Score (95% CI)	F1 score (95% CI)	Accuracy (95% CI)
Model 1	0.01 (0.00-0.02)	0.04 (-0.01-0.09)	0.04 (-0.01- 0.08)
Model 2	0.01 (0.00-0.02)	0.05 (0.01-0.09)	0.04 (0.00-0.08)
Model 3	0.01 (0.00-0.02)	0.01 (0.02-0.04)	0.01 (0.02-0.04)

Abbreviations: ASPECTS, the Alberta Stroke Program Early Computed Tomography Score; CI, confidence interval; EVT, endovascular thrombectomy; HGI, HbA1c glycation index; NIHSS, National Institute of Health Stroke Scale; PTR, from puncture to recanalization; WBC, white blood cell count.

Model 1: adjusted for age and sex.

Model 2: additionally adjusted for smoke, drink, hypertension, diabetes mellitus, hyperlipidemia, coronary heart disease, and atrial fibrillation.

Model 3: adjusted for age, diabetes mellitus, PTR, baseline NIHSS score, baseline ASPECTS score, number of attempts, HbA1c, WBC, neutrophil count and lymphocyte count.

Table 3. Association between HGI and Clinical Outcomes after EVT in patients with successful recanalization.

	Model 1		Model 2		Model 3	
**Outcomes**	**OR (95% CI)**	***P* value**	**OR (95% CI)**	***P* value**	**OR (95% CI)**	***P* value**
Poor outcome						
HGI Tertile 1 vs. 2	2.88 (1.68-5.02)	0.001	3.11 (1.78-5.51)	0.001	3.67 (1.96-7.02)	0.001
HGI Tertile 3 vs. 2	1.96 (1.15-3.38)	0.015	1.31 (0.71-2.39)	0.388	1.35 (0.63-2.90)	0.438
END						
HGI Tertile 1 vs. 2	2.11 (1.08-4.26)	0.033	2.32 (1.16-4.78)	0.019	3.07 (1.49-6.59)	0.003
HGI Tertile 3 vs. 2	2.13 (1.09-4.32)	0.030	1.93 (0.92-4.16)	0.086	1.42 (0.61-3.33)	0.419
ICH						
HGI Tertile 1 vs. 2	1.58 (0.87-2.89)	0.134	1.58 (0.87-2.94)	0.138	1.65 (0.89-3.11)	0.114
HGI Tertile 3 vs. 2	1.30 (0.70-2.41)	0.405	1.09 (0.55-2.16)	0.796	1.06 (0.49-2.29)	0.877
SICH						
HGI Tertile 1 vs. 2	1.98 (0.89-4.63)	0.100	2.22 (0.98-5.32)	0.063	2.68 (1.15-6.59)	0.025
HGI Tertile 3 vs. 2	1.86 (0.83-4.41)	0.141	1.49 (0.61-3.79)	0.388	1.24 (0.46-3.42)	0.674

Abbreviations: ASPECTS, the Alberta Stroke Program Early Computed Tomography Score; CI, confidence interval; EVT, endovascular thrombectomy; HGI, HbA1c glycation index; NIHSS, National Institute of Health Stroke Scale; OR, odds ratio; PTR, from puncture to recanalization; WBC, white blood cell count.

Model 1: adjusted for age and sex.

Model 2: additionally adjusted for smoke, drink, hypertension, diabetes mellitus, hyperlipidemia, coronary heart disease, and atrial fibrillation.

Model 3: adjusted for age, diabetes mellitus, PTR, baseline NIHSS score, baseline ASPECTS score, number of attempts, HbA1c, WBC, neutrophil count and lymphocyte count.

Table 4. Association between HGI Calculated Using the New Formula and Clinical Outcomes after EVT.

	Model 1		Model 2		Model 3	
**Outcomes**	**OR (95% CI)**	***P* value**	**OR (95% CI)**	***P* value**	**OR (95% CI)**	***P* value**
Poor outcome						
HGI Tertile 1 vs. 2	3.29 (1.63-6.93)	0.001	3.15 (1.53-6.79)	0.002	4.03 (1.78-9.57)	0.001
HGI Tertile 3 vs. 2	1.14 (0.72-1.79)	0.578	0.81 (0.49-1.33)	0.410	0.78 (0.42-1.44)	0.424
END						
HGI Tertile 1 vs. 2	1.54 (0.70-3.26)	0.269	1.51 (0.67-3.28)	0.305	1.59 (0.70-3.53)	0.257
HGI Tertile 3 vs. 2	1.30 (0.75-2.28)	0.356	1.16 (0.64-2.13)	0.632	0.76 (0.39-1.49)	0.426
ICH						
HGI Tertile 1 vs. 2	1.87 (0.94-3.69)	0.072	1.67 (0.83-3.35)	0.147	1.96 (0.96-3.97)	0.063
HGI Tertile 3 vs. 2	1.05 (0.63-1.77)	0.842	0.97 (0.56-1.71)	0.926	0.94 (0.51-1.76)	0.857
SICH						
HGI Tertile 1 vs. 2	1.94 (0.81-4.51)	0.129	1.88 (0.76-4.53)	0.165	2.00 (0.80-4.83)	0.128
HGI Tertile 3 vs. 2	1.21 (0.62-2.41)	0.579	1.00 (0.48-2.12)	0.997	0.79 (0.35-1.77)	0.561

Abbreviations: ASPECTS, the Alberta Stroke Program Early Computed Tomography Score; CI, confidence interval; EVT, endovascular thrombectomy; HGI, HbA1c glycation index; NIHSS, National Institute of Health Stroke Scale; OR, odds ratio; PTR, from puncture to recanalization; WBC, white blood cell count.

Model 1: adjusted for age and sex.

Model 2: additionally adjusted for smoke, drink, hypertension, diabetes mellitus, hyperlipidemia, coronary heart disease, and atrial fibrillation.

Model 3: adjusted for age, diabetes mellitus, PTR, baseline NIHSS score, baseline ASPECTS score, number of attempts, HbA1c, WBC, neutrophil count and lymphocyte count.
